# Accuracy of parasitological techniques and positivity for endoparasites in stray and household cats in Jataí, Goiás, Brazil

**DOI:** 10.1590/S1984-29612025010

**Published:** 2025-04-07

**Authors:** Isa Marianny Ferreira Nascimento Barbosa de Souza, Stéfanne Rodrigues Rezende Ferreira, Samuel Felipe Netzlaff, Amanda Cristina Corrêa Fleury, Victor da Silva Siqueira, Gabriella Katrinny Avelar Oliveira, Isabella da Costa Ribeiro, Júlia Batista Dornelas, Ludimila Paula Vaz Cardoso, Hanstter Hallison Alves Rezende

**Affiliations:** 1 Laboratório de Bacteriologia e Micologia, Instituto de Ciências da Saúde, Universidade Federal de Jataí – UFJ, Jataí, GO, Brasil

**Keywords:** Parasitological techniques, Felis catus, cats, endoparasites, Técnicas parasitológicas, Felis catus, gatos, endoparasitos

## Abstract

The objective of this study was to describe the positivity of intestinal parasitosis in cats from the city of Jataí, Goiás, as well as to determine the accuracy of different parasitological techniques applied. 120 samples were collected, 64 from household cats and 56 from stray. The parasitological techniques of Willis, Sheather, Faust, Hoffman-Pons-Janer-Lutz and Rugai staining were then performed. The positivity rate of endoparasites in the analyzed samples was 19.2%, with a predominance of hookworm eggs. *Cystoisospora* spp. and *Hammondia hammondi* oocysts were also found, with a case of co-infection with the two species. No statistical difference in positivity was found between household and stray cats. However, a significant difference was found upon evaluating sex and neutering status, revealing that male cats and non-neutered animals showed higher positivity rates, with 28.8% of non-neutered animals testing positive compared to 9.83% of neutered animals. The Willis technique was considered the gold standard for hookworm diagnosis, with a sensitivity of 91.3%. While the Willis technique was identified as the gold standard for detecting hookworm eggs, the evaluation of other methodologies demonstrated that the integration of techniques can improve the detection of various parasitic stages.

## Resumo

O objetivo deste trabalho foi descrever a positividade de parasitoses intestinais em gatos da cidade de Jataí, Goiás, bem como determinar a acurácia das diferentes técnicas parasitológicas aplicadas. Foram coletadas 120 amostras fecais, sendo 64 de gatos domiciliados, e 56 de errantes. Em seguida, foram realizadas as técnicas parasitológicas de Willis, Sheather, Faust, Hoffman-Pons-Janer-Lutz e Rugai. A positividade para enteroparasitos nas amostras analisadas foi de 19,2%, sendo, predominantemente, encontrados ovos de ancilostomídeos. Além disso, foram identificados oocistos de *Cystoisospora* spp. e de *Hammondia hammondi*, com um caso de coinfecção entre estes. Ademais, não houve diferença estatística na positividade entre gatos domiciliados e errantes. No entanto, uma diferença significativa foi encontrada ao avaliar o sexo e o estado de castração. Essa observação revelou que gatos machos e animais não castrados apresentaram maiores taxas de positividade, com 28,8% dos animais não castrados que apresentaram resultados positivos, em comparação com 9,83% dos animais castrados. A técnica de Willis foi considerada padrão-ouro para o diagnóstico de ancilostomídeos, apresentando sensibilidade de 91,3%. Embora a técnica de Willis tenha sido identificada como padrão-ouro para a detecção de ovos de ancilostomídeos, a avaliação de outras metodologias demonstrou que a integração de técnicas pode melhorar a detecção de diversos estágios parasitários.

## Introduction

The domestication of animals, particularly the domestic cat (*Felis catus*), has significantly increased human contact with species that were once wild. This close relationship has raised public health concerns, as cats can serve as hosts for a variety of parasites, including helminths and protozoa, which can be transmitted to humans through contaminated water or food sources (Recht et al., 2020).

Among the main zoonotic parasites found in cats, protozoa like *Giardia* spp., *Cryptosporidium felis*, and *Toxoplasma gondii* are notable. While *Giardia* spp. and *C. felis* cause gastrointestinal symptoms, and *T. gondii* is particularly concerning due to its high positivity and potential to cause severe conditions in immunocompromised individuals, such as those with HIV (Andrade et al., 2012).

In addition to protozoa, helminths are also of significant concern, especially in terms of public health. For example, Ancylostomatidae (hookworms) are commonly found in cats and present a risk for humans through the transmission of cutaneous larva migrans, particularly in environments like parks and beaches where people, especially children, may come into contact with contaminated soil (Silva et al., 2017).

Studies have shown a high positivity of helminth infections in stray cats, reflecting their increased exposure to unsanitary environments and lack of routine veterinary care. For instance, surveys conducted in Brazil indicate varied positivity rates of helminths in cats, with estimates ranging from 12.5% to 83.5% across different regions, highlighting the widespread presence of these parasites in both stray and household cats (Rezende et al., 2015; Silva et al., 2017; Ferraz et al., 2019; De Paula et al., 2021).

Understanding the positivity for endoparasite infections in cats is essential to assess the risk of zoonotic disease transmission to humans. Cats that live in hygienic conditions, with adequate housing and diet, are generally less exposed to parasitic infections, thus reducing their potential to act as sources of zoonotic transmission. However, there remains a scarcity of data comparing the positivity of endoparasites in stray and household cats in Brazil, especially in the southwest region of Goiás, which limits our understanding of the factors that may poinfluence infection rates in different cat populations (Ramos et al., 2022).

This study aims to address this gap by investigating the positivity for intestinal parasites in adult cats and kittens in the city of Jataí, Goiás. In addition, the study seeks to examine how factors such as sex and reproductive status affect infection rates and to evaluate the accuracy of various parasitological techniques used for diagnosing these infections.

## Materials and Methods

### Study design

The cats were divided into two groups: stray and household. The household animals were included in the study through direct contact with their owners, while the stray animals were provided by non-governmental organizations (NGOs) that rescue stray animals.

A total of 120 samples were collected between June and December 2021, 64 from household cats and 56 from stray cats. The samples were delivered to the Laboratory of Clinical Biochemistry and Body Fluids of UFJ, where they were subjected to parasitological analysis.

### Parasitological analysis

The samples were examined in order to identify helminth eggs and larvae, as well as protozoan cysts and oocysts, using the following parasitological techniques: spontaneous flotation in a sodium chloride solution, with a density of 1.2 g/mL (Willis, 1921); centrifugal flotation in 33% zinc sulfate, density 1.18 g/mL (Faust et al., 1938); centrifugal flotation in sucrose, density 1.2 g/mL (Sheather, 1923); and spontaneous sedimentation (Hoffman et al., 1934). In addition, the Rugai technique (Rugai et al., 1954) was also performed to identify helminth larvae, and the Ziehl-Neelsen staining method was applied to the sediment to detect protozoan oocysts.

### Copro-PCR protocol for detecting *Toxoplasma gondii*

DNA was extracted from the fecal samples using the commercial mini kit for genomic DNA purification from blood (Ludwig Biotec), following the manufacturer's guidelines. To facilitate the disruption of the oocyst wall, the samples were treated with a lysis buffer, boiled for 20 minutes, and incubated overnight at 37 °C. The DNA extraction process was completed the following day.

Polymerase chain reactions (PCR) were conducted in a reaction volume of 25 μL. The mixture contained 10 mM TRIS HCl (pH 9.0), 3.5 mM MgCl2, 0.2 U of Taq DNA Polymerase (Invitrogen), 0.5 mM of each dNTP (Sigma Chemical Co., USA®), 50 pmol of each primer (Invitrogen®), and 5 μL of the extracted DNA template. The reactions were run on a MasterCycler Personal thermal cycler using the following program: initial denaturation at 94 °C for 5 minutes, followed by 35 cycles of denaturation at 94 °C for 1 minute, annealing at 62 °C for 1 minute, and extension at 72 °C for 1 minute. A final extension was performed at 72 °C for 10 minutes.

The primers used included Toxo-B5 and B6 and TOX4 and 5, which amplify a 529 bp fragment. Positive controls consisted of mouse peritoneal fluid infected with RH and ME49 strains of *T. gondii*, along with previously confirmed positive fecal samples. Negative controls consisted of DNA extracted from fecal samples previously verified as free of *T. gondii*. PCR products (110 bp) were analyzed by electrophoresis on a 6% polyacrylamide gel, stained with silver.

### Statistical analysis

An evaluation was made of the positivity of endoparasites in samples, as well as the positivity rates achieved with each of the techniques. The parameters of sensitivity, specificity, positive predictive value (PPV), negative predictive value (NPV) and Kappa (K) of the techniques were determined by comparison with the gold standard technique (Willis). The confidence adopted in the study was 95%. The *X^2^* test was performed to correlate positivity in household and stray animals, male/female and neutered/non-neutered. These results were obtained and analyzed using Microsoft Excel® 2013 and BioStat® 5.3. The accuracy of the techniques was analyzed based on the most prevalent parasite, comparing each method with the Willis technique (gold standard). The low positivity value adopted was less than 5%. Average positivity was defined as being between 5% and 10%, while high positivity was defined as above 10%.

## Results and Discussion

A total of 120 samples were collected, 64 from household cats and 56 from stray cats. Among these samples, 19.2% (23/120) were tested positive for endoparasites, 9 samples from household animals and 14 from strays. Thus, the positivity rate was 14% (9/64) among household cats, and 25% (14/56) among stray cats.

The *X^2^* test, which was applied to compare the positivity rates of household and stray cats, indicating no statistical difference (p=0.13). However, Lima et al. (2018), who analyzed fecal samples from cats in the city of Goiânia, state of Goiás, Brazil, found a higher and statistically significant positivity of endoparasites in stray animals. It is worth noting that the behavior of household cats, such as potential outdoor access and interactions with other animals, could influence their exposure to endoparasites, potentially reducing the expected differences in positivity between the two groups. A study conducted in the city of São Paulo also found higher parasitism among stray cats, which was attributed to lack of antiparasitic treatment and malnutrition (Serra et al., 2003).

It is important to acknowledge that the sample size of stray cats in this study may have influenced the results. While no significant difference in positivity rates was observed between stray and household cats, the observed trend of higher positivity in stray cats suggests that further studies with larger sample sizes are needed to confirm these findings and explore the potential factors influencing infection rates in these populations.

On the other hand, inadequate antiparasitic treatment, as well as unrestricted contact between household and stray animals, can favor transmission, increasing positivity among household cats, which corroborates the findings reported by Ferreira et al. (2013). A study by Lima et al. (2021) in Santos, SP, demonstrated high endoparasite positivity rates in household cats, highlighting the importance of treatment and early diagnosis of these companion animals. It is worth noting that the appropriate parasitic treatment is carried out with a protocol defined by the veterinarian based on a final diagnosis.

A comparison of positivity rates was conducted among cats, considering the factors of sex and spaying/neutering. Using the chi-square test (X^2^), it was found that male cats had a significantly higher positivity rate for endoparasites, at 29.2% (19/65), compared to females, who exhibited a positivity rate of 7.3% (p < 0.05). This significant difference can be attributed to behavioral patterns, as male cats typically roam greater distances and leave their homes more frequently to mark their territories, increasing their exposure to contaminated environments and, consequently, their risk of infection with endoparasites (Ellis et al., 2017).

As for spaying/neutering, a higher positivity rate was found among non-neutered animals (28.8%; 17/59) than among neutered animals (9.83%; 6/61), with *p*= 0.01. It is known that the sterilization of cats can reduce the propensity of these animals to roam outside their home, significantly contributing to reduce endoparasite infections (Machado et al., 2018).

Among the animals that tested positive, 16.7% (20/120) were infected with Ancylostomatidae, two with *Cystoisospora* spp., 1.7% (2/120) and 1 with polyparasitism by *Cystoisospora* spp. and *H. hammondi* 0.8% (1/20). The predominance of infection by Ancylostomatidae has been observed in other studies performed on cat feces in different regions of the country (Ferraz et al*.,* 2021; Lima et al.*,* 2018; Rezende et al., 2015; Serra et al.*,* 2003).

The high positivity for Ancylostomatidae constitutes a public health concern, since individuals, especially children, are exposed to possibly contaminated environments such as beaches, public parks, and school areas, increasing the likelihood of acquiring cutaneous larva migrans (CML) (Andrade et al.*,* 2012; Silva et al.*,* 2017). In addition, Ancylostomatidae infections can last or be repeated throughout the animal’s life, since they do not depend on acquired immunity (Monteiro et al., 2016).

Epidemiological studies in Brazil have identified a variety of positivity rates for Ancylostomatidae in cats ranging from 83.5% (96/115) to 56% (14/25), 12.5% (2/10) and to 65% (10/15) in the states of Goiás, Rio Grande do Sul, Minas Gerais and Maranhão, respectively (Rezende et al., 2015; Silva et al., 2017; Ferraz et al., 2019; De Paula et al., 2021). The predominance of Ancylostomatidae may be associated with the monoxenous life cycle and high egg production by females (Pereira et al., 2017).

Only two samples (1.7%; 2/120) showed monoparasitism by *Cystoisospora* spp., which brings to mind the findings of Lima et al. (2018) in Goiás, where only one sample was positive for monoparasitism (2.6%; 1/39).

Furthermore, 1 out of 120 samples (0.8%) was positive for *H. hammondi*, unlike the study of Igreja et al. (2021), who reported a positivity rate of 15.4% (21/115) in Goiânia, Goiás. When an *H. hammondi* oocyst is found in a parasitological fecal test, the *T. gondii* B1 gene is differentiated by means of polymerase chain reaction (PCR) and/or REP-529 sequence. In this study, the two aforementioned techniques were negative for *T. gondii*, confirming the diagnosis of *Hammondia* sp. Therefore, there was no positive sample for *T. gondii*, which differs from the findings of Rezende et al. (2015).

The positivity rates achieved by the diagnostic techniques were evaluated based on the positive samples. A 91.3% (21/23) positivity rate was obtained using the Willis technique, with only two false negative results; hence, it was considered the gold standard for accuracy analysis (Rezende et al., 2015; Lima et al., 2018). False negative results were verified with two samples that were positive only in the Sheather technique. The Sheather and Faust methods produced the lowest positivity rates, both with 13% (3/23), with the detection of only one positive sample. A positivity rate of 26.1% (6/23) was obtained by the HPJL technique. On the other hand, the Rugai technique was considered inaccurate, since only one sample was found to be positive, based on the detection of oocysts identified as *Cystoisospora* spp., probably because the animal exhibited a high level of parasitism.

These results differ from those reported in other studies. Lima et al. (2018) observed a greater correlation when combining the Willis and Faust techniques, reaching 75% sensitivity and kappa index of 0.805. On the other hand, Rezende et al. (2015) found a greater correlation between the Willis and Sheather techniques, which reached 81% sensitivity. However, these two cited studies presented higher positivity rates than those of the current research.

Based on the results obtained here, it can be stated that the three techniques have good specificity for diagnosing light helminth eggs such as those of Ancylostomatidae. Among the methods analyzed here, the Sheather and Faust techniques exhibit limitations in the detection of helminth eggs.

The analysis of accuracy of the Sheather and HPLJ techniques in the diagnosis of *Cystoisospora* spp. showed similar results, i.e., 33.3% sensitivity, 100% specificity and kappa index of 0.494, with a good correlation. The Faust technique was unsuccessful in the diagnosis of the parasite in any samples, demonstrating 0% sensitivity and 100% specificity, without a calculation of the kappa index. The studies by Lima et al. (2018) and Rezende et al. (2015) also found a good correlation between the Sheather and HPLJ techniques in the diagnosis of *Cystoisospora* spp.

These results indicate that the Sheather and HPLJ techniques are reliable for the diagnosis of oocysts. Additionally, in other studies, Sheather’s method has proven effective for the detection of protozoan cysts, such as those of *Giardia* spp*.*, and oocysts, such as those of *Cryptosporidium* spp., highlighting its versatility in identifying different types of parasitic stages (Kamrani et al., 2021).

In short, the analyzed samples showed a predominance of Ancylostomatidae. This means that educational actions among the general population would be very useful, focusing especially on owners of household pets, to clarify problems pertaining to zoonotic diseases and preventive precautions they can adopt. Such measures include periodic removal of feces, restricted contact with animals living in potentially infected environments, as well as the correct administration of antiparasitic drugs, following professional advice.

The Willis technique was considered the gold standard in this study due to its effectiveness in detecting positive samples. This method, which utilizes spontaneous flotation in a sodium chloride solution, demonstrated superior sensitivity in identifying parasitic eggs. In contrast, other techniques, such as Sheather’s sugar flotation, Faust’s zinc sulfate flotation, and the Rugai method, showed limited effectiveness and a lower level of agreement with the gold standard, indicating variability in diagnostic performance.
